# Mischievous Odds Ratios

**DOI:** 10.1371/journal.pmed.0030205

**Published:** 2006-04-25

**Authors:** William Steinsmith

**Affiliations:** **1**Institute of Thermodynamic Biology and MedicineWilliamsburg, VirginiaUnited States of America

Pieter Reitsma and colleagues have explored—in a population of patients anticoagulated with coumarin congeners—the connection between the presence of mutant alleles of a single gene and the risk of haemorrhage [ 1].

Using as their denominator the odds for bleeding in a patient without mutant alleles, and using as their numerator the odds for patients with each of the two mutant alleles, the authors propose the resulting odds ratios as surrogates for the relative risk of haemorrhage.

It should be noted, however, that the conflation of an odds ratio with a relative risk is not generally justified [ 2, 3]. The relative risk is the ratio of two probabilities (p2/p1), whereas the corresponding odds ratio is [p1/(1-p1)]/[p2/(1-p2)]. Equating these two ratios requires that p1 = p2, i.e., that the risk ratio be unity.

In Reitsma and colleagues' paper, none of the eight odds ratios presented in Table 2 turn out identical with the corresponding calculated risk ratio, and the most discordant pair of values diverge by a factor of about 1.4, i.e., the odds ratio of 2.6 corresponding to a relative risk of 1.9.

Mischievous conflation of odds ratios with probability ratios is widespread in the literature dealing with laboratory testing, with the odds ratio (confusingly termed the “likelihood ratio”) typically presented as surrogate for the corresponding ratio of probabilities.

The power of a positive laboratory test to enhance the likelihood of disease presence in a given patient (properly termed the “positive probability-based likelihood ratio”) is the ratio of two probabilities: the probability that the patient who tested positive is truly diseased (termed the “positive predictive value”) divided by the probability of disease in the pre-test population (termed the “disease prevalence”).

Expressed explicitly in terms of the subcategories of the test population, the positive predictive value is the ratio represented by (True Positives)/(True Positives + False Positives), and the prevalence is the ratio represented by (True Positives + False Negatives)/(True Positives + False Negatives + True Negatives + False Positives).

The calculus is easily adapted to compute the probability-based likelihood ratio for the absence of disease in a given patient. In this case, the post-negative-test probability of disease absence (termed the “negative predictive value”) is the ratio represented by (True Negatives)/(True Negatives + False Negatives), and the pre-test probability is one minus the disease prevalence. The negative probability-based likelihood ratio is, then, the ratio represented by the post-test probability divided by the pre-test probability.

A more descriptive term for the probability-based likelihood ratio would be the “probability magnifying power,” since it leads to the expanded probability of the presence (or absence) of disease yielded by a positive (or negative) test result.
